# Presentation, surgery and 1-year outcomes of childhood cataract surgery in Tanzania

**DOI:** 10.1136/bjophthalmol-2020-316042

**Published:** 2020-06-10

**Authors:** Furahini Godfrey Mndeme, Blandina Theophyl Mmbaga, Mchikirwa Msina, Judith Mwende, Sonia J Vaitha, Min J Kim, David Macleod, Matthew J Burton, Clare E Gilbert, Richard Bowman

**Affiliations:** 1 Ophthalmology, Kilimanjaro Christian Medical University Collage, Moshi, Tanzania, United Republic of; 2 Clinical Research, London School of Hygiene and Tropical Medicine, London, UK; 3 Paediatrics, Kilimanjaro Clinical Research Institute, Kilimanjaro Christian medical Centre, Moshi, Tanzania, United Republic of; 4 Ophthalmology, Muhimbili National Hospital, Dar-Es-Salaam, Dar-Es-Salaam, Tanzania, United Republic of; 5 Ophthalmology, Comprehensive Community Based Rehabilitation in Tanzania, Dar-Es-Salaam, Tanzania, United Republic of; 6 Tropical Epidemiology Group, Faculty of Infectious Disease Epidemiology, London School of Hygiene and Tropical Medicine, London, London, UK; 7 Medical Statistics, London School of Hygiene & Tropical Medicine, London, UK; 8 Ophthalmology, Great Ormond Street Hospital, London, UK

**Keywords:** child health (paediatrics), lens and zonules, treatment surgery

## Abstract

**Background:**

Recent reports have suggested a significant change in the causes of blindness in children in low-income countries cataract becoming the leading cause. We aimed to investigate the presentations and surgical outcomes in children with cataract operated at different ages in Tanzania.

**Methods:**

We conducted a prospective study of 228 children aged ≤192 months at three tertiary centres, 177 with bilateral cataracts and prospectively followed them for 1-year postsurgery. We collected demographic, surgical, preoperative and postoperative clinical characteristics using the standard childhood cataract surgical assessment questionnaire. Families were encouraged to return for follow-up by phone with travel reimbursement where necessary.

**Results:**

Preoperatively, 76% bilateral children were blind in the better eye. 86% of children were followed up at 1 year and 54% bilateral children achieved visual acuity of 0.48 logMAR or better in the better eye and 5% were blind. 33% of unilateral children achieved visual acuity of 0.48 logMAR or better and 17% were blind. Preoperative blindness (adjusted OR (AOR) 14.65; 95% CI 2.21 to 97.20), preoperative nystagmus/strabismus (AOR 9.22; 95% CI 2.66 to 31.97) and aphakia (AOR, 5.32; 95% CI 1.05 to 26.97) predicted poor visual outcome in bilateral cases. 9% of 342 refracted eyes had initial postoperative cylinder of 1.5 D or more, as did a similar proportion (11%) of 315 eyes refracted 1 year after surgery. Acute fibrinous uveitis occurred in 41 (12%) eyes.

**Conclusion:**

Three-quarters of children were blind preoperatively whereas over half had good vision 1-year postoperatively. Preoperative blindness, nystagmus/strabismus and aphakia predicted poor visual outcome, suggesting that cataract density determines density of amblyopia.

## Introduction

Successful measles immunisation and vitamin A supplementation have resulted in a fall in the prevalence of blindness in children in Sub-Saharan Africa (SSA), from approximately 1 per 1000 to 0.6 per 1000.^1^ Recent research has suggested a significant change in the causes of blindness in children in low-income countries, with cataract becoming the leading avoidable cause.^2 3^

Visual outcomes are poorer following surgery for bilateral cataract in SSA, than in high-income countries. Studies in SSA show that 8%–60% of children achieve best-corrected visual acuity of 0.48 logMAR or better after surgery^4–6^ compared with 72%–91% in studies undertaken in high-income settings.^7 8^ Possible reasons for the variation include late presentation, higher surgical and postoperative complication rates, poor follow-up and inadequate correction of aphakia.^5 6 9^ However, studies in SSA and lower-middle-income countries are potentially biased due to low follow-up rates. To date, there are no reliable prospective reports of outcomes of congenital cataract in SSA with good follow-up. The aim of this prospective study was to describe visual outcomes and the causes and risk factors for poor outcomes in children with bilateral and unilateral cataract operated at different ages Tanzania.

## Materials and methods

### Participant recruitment

This was a prospective, longitudinal hospital-based observational study of children undergoing cataract surgery who were recruited between November 2016 and August 2017. Children aged <16 years were recruited from three tertiary facilities in Tanzania. All children underwent preliminary evaluation for study eligibility by paediatric ophthalmologists (FGM, JM, and SJV). We excluded children with traumatic cataracts and minor lens opacities not requiring surgery. Sociodemographic and clinical details were recorded by investigating clinicians using a questionnaire. Further data were collected to investigate risk factors for cataract. Weight and haemoglobin at surgery were compared with published national averages.^10 11^

Visual acuity in children 3 years and below was assessed with the Teller Acuity Cards. Children aged 4–7 years were initially assessed usingHOTV matching method, and if this was not possible, Lea Symbols were used, and failing this, Teller acuity cards were used. Children aged 8 years and above were tested using Snellen Charts. All results were converted to logMAR values. Cataracts were examined by direct ophthalmoscopy and slit lamp through dilated pupils. Cataract free lens and the total cataract were subjectively graded as grade 0 and 10, respectively. Cataracts with static morphologies^12^ were assigned fixed grades (ie, grade 1 for anterior polar and sutural cataracts, and grade 6 for posterior polar cataracts). Progressive cataracts were given grading ranges, indicating the initial and likely final morphology states.^12^

Biometry was performed in the operating room after induction of general anaesthesia. Intraocular lens (IOL) power was chosen to be approximately 20% less than that predicted to achieve emmetropia in children younger than 2 years; a 10% under correction for children from 2 to 8 years of age and emmetropia for older children.^13^ In children for whom keratometry reading were not obtained, axial length alone was used.^14^

Four surgeons conducted surgeries; posterior capsulotomy and anterior vitrectomy were performed for children less than 7 years of age using an Alcon Accurus vitrectomy machine via an anterior approach or through a pars plana incision. In children above 1 year of age, the main corneal wound and paracenteses sites were sealed with hydroinflation; in children under 1 year both were closed with 9–0 nylon sutures. Individual surgeons used their standard technique.

IOLs were routinely implanted under Aurogel (sodium hyaluronate 1.4%) for children aged 4 months and above, with exceptions being a corneal diameter of <9.5 mm; or an axial length of <17 mm or other ocular abnormalities, which precluded implantation. Alcon AcrySoft single-piece and multi-piece IOLs (Alcon Laboratories, Fort Worth, Texas, USA) were used routinely according to availability for all children during the study period. At the end of the procedure, intracameral ceftriaxone (1 mg), and subconjunctival dexamethasone (2 mg) and gentamycin (10 mg) were administered. Subconjunctival triamcinolone-acetate (20 mg) was administered to patients at high risk of uveitis.

All children were examined on the first day and stayed in hospital for 1 week. They were then followed up at 2 weeks, 1 month, 3 months, 6 months and 1 year postoperatively and visual acuity, refractive error and intraocular pressure (IOP) were measured at each follow-up and postoperative complications were recorded. Refraction was first performed 1 week postoperatively and when needed, spectacles were given to all children regardless of age. IOP was measured using I-Care and a standard proforma was used to record all postoperative findings. Parents were counselled at discharge and at all other visits about the importance of follow-up. Contact details were recorded to allow for reminders of subsequent follow-up and travel expenses were provided, if required.

Statistical analysis was conducted using STATA V.14. Descriptive analysis to determine the frequency of postoperative refractive errors and visual acuity improvement over time was performed. All plausible predictors of visual outcome were tested by univariate analyses and those found to have association with the outcome (at p<value less than 0.05) were further analysed in a multivariate model.

There was an opportunity to discuss and ask questions. Finally, if the parent or guardian agreed to allow the child to be enrolled into the study, this was documented on a consent form in Kiswahili, and witnessed by a third person.

## Results

### Demographics

A total of 228 children (177 (78%) with bilateral cataracts and 51 (22%) with unilateral cataracts, 405 eyes total) aged ≤192 months (mean age 41.1 months, median 26 months, IQR 10–60) were recruited. One hundred and forty-one (61.8%) were male; 111 (62.7%) with bilateral cataracts and 30 (58.8%) with unilateral cataracts). Eighty-seven (38%) were younger than 1 year at presentation and 164 (72%) were younger than 6 years. Sixty-nine out of 177 (38.9%) bilateral children were younger than 1 year old and were the subject of a separate case control study on risk factors. Description of patients by laterality according to age, sex, cataract types and tertiary care facility are shown in table 1.

**Table 1:** Description of patients by laterality according to age, sex, cataract types and tertiary care facility

Characteristics		Bilateral N (%)	Unilateral N (%)	Total n (%)	Cumulative Frequency
Age group (months)	≤3	9 (5.1)	3 (5.9)	12 (5.3)	5.3
	4–6	15 (8.5)	5 (9.8)	20 (8.7)	14.0
	7–12	45 (25.4)	10 (19.6)	55 (24.1)	38.1
	13–18	8 (4.5)	2 (3.9)	10 (4.4)	42.5
	19–24	13 (7.3)	4 (8.8)	17 (7.5)	50.0
	25 and above	87 (49.2)	27 (52.9)	114 (50)	100.0
	**Total**	**177(100**)	**51(100**)	**228(100**)	
Sex	Male	111 (62.7)	30 (58.8)	141 (61.8)	61.8
	Female	66 (37.3)	21 (41.2)	87 (38.2)	100.0
	**Total**	**177 (100**)	**51 (100**)	**228 (100**)	
Tertiary care facility	KCMC	102 (57.6)	21 (41.2)	123 (53.9)	53.9
	CCBRT	49 (27.7)	30 (58.8)	79 (34.6)	88.6
	MNH	26 (14.7)	0 (0.0)	26 (11.4)	100.0
	**Total**	**177 (100**)	**51 (100**)	**228 (100**)	
Cataract types	No of eyes				
	Diffuse/total	130 (36.7)	30 (58.8)	160 (39.5)	39.5
	Nuclear	86 (24.3)	5 (9.8)	91 (22.5)	62.0
	Lamellar	129 (36.4)	10 (19.6)	139 (34.3)	96.3
	PHPV	0 (0.0)	2 (3.9)	2 (0.5)	96.8
	Anterior polar	0 (0.0)	2 (3.9)	2 (0.5)	97.3
	Posterior polar	5 (1.4)	1 (1.9)	6 (1.5)	98.8
	Cortical	2 (0.6)	1 (1.9)	3 (0.7)	99.5
	Membranous	2 (0.6)	0 (0.0)	2 (0.5)	100.0
	**Total**	**354** (**100**)	**51** (**100**)	**405** (**100**)	

CCBRT, Comprehensive Community Based Rehabilitation in Tanzania; KCMC, Kilimanjaro Christian Medical University College; MNH, Muhimbili National Hospital; PHPV, Persistent Hyperplastic Primary Vitreous.

Body weight at surgery was lower (mean 13.3 (SD; 7.1) kg) in cataract patients compared with the age-matched census data for normal babies and teenagers^15^ (mean 15.1 (SD; 8.6) kg (p=0.01). This was more common (p=0.02) in bilateral cases 111/177 (62.7%) than unilateral 24/51 (47.1%). Sixty-four of 177 (36.2%) bilateral children had low birth weight compared with 7 of 51 (13.7) unilateral children (p=0.001). Eighteen of 177 (10.2%) children with bilateral cataract were born prematurely (before 37 weeks) compared with one of 51 (2.0%) children with unilateral cataract (p=0.03). Twenty-one (9.2%) children had a family history, more commonly (p=0.06) bilateral 19/177 (10.9%) than unilateral 2/51 (3.9%). Anaemia was also associated (p=0.05) with bilaterality 89/177 (52.3%) vs unilaterality 19/51 (37.2%). Anaemia was also associated with denser cataracts (grade 8 and above) (p=0.01) and preoperative blindness (p=0.03).

### Associated motility disturbance

Preoperative findings are shown in online supplementary table 1. Based on clinical judgement, nystagmus disappeared after surgery in 13/96 (13.5%) children. Children who were blind preoperatively were less likely (p=0.01) to show improvement in nystagmus.

10.1136/bjophthalmol-2020-316042.supp1 Supplementary data

### Lag time (time between carers noticing a problem and cataract surgery)

The mean was 21.0 months (SD: 26.7 months, range: 0–133 months), with 115 (52.5%) children having ≥12 months. Of 177, 127 (71.8%) bilateral children had lag time of 6 months and above compared with 30/51 (58.8%) children (p=0.04) with unilateral cataracts. There was no significant difference in lag time by gender (online supplementary table 2).

### Surgery

There was a change of surgical technique for two surgeons (FGM and SJV) during the study, from 3 mm superior scleral tunnel incision (sutured) to 3 mm (usually sutureless) superior corneal incision and corneal paracenteses at 3 and 9 o’clock; no wound leaks, endophthalmitis or shallow anterior chambers developed. A total of 249 (61.5%) surgeries were done through corneal incisions and 61.8% of all surgeries were completely sutureless. All wounds in children less than 1 year, and scleral-tunnels and sclerostomies in any age were sutured.

IOLs were inserted in 346 of 405 (85.4%) eyes operated: AcrySoft single-piece 255 (62.9%) and three-piece lenses 91 (22.5%). Intracapsular fixation of the IOL was achieved in 290 (71.4%) eyes and 56 (13.8%) were sulcus fixated. A total of 390 (96.1%) eyes underwent posterior capsulotomy and anterior vitrectomy, in 142 (34.9%) eyes via a pars plana approach and 259 (63.8%) eyes via an anterior approach (missing data in five eyes).

### Follow up and complications

One hundred and fifty-four out of 177 (87.0%) children with bilateral cataracts and 42 out of 51 (82.4%) children with unilateral cataracts were followed up at 1 year postoperatively. Two children died before 1-year follow-up, one after a month and one after 6 months follow-up. No child died during the perioperative period.

Postoperative complications and reoperation rates by laterality are shown in table 2. In univariate analysis, younger age at surgery (OR=0.: p=0.011) and multi piece IOL fixation (OR=3.0: p=0.019) were associated with post-operative fibrinous uveitis, but in a multivariate model, only multipiece IOL remained significant (adjusted OR=2.6: p=0.05).

**Table 2:** Postoperative complications and reoperation rates at 1 year by laterality (n=350 eyes)

Characteristics	Bilateral cataract n (%)	Unilateral cataract n (%)	Total n (%)
**Early surgical complications**			
Acute fibrous uveitis	35 (11.4)	6 (14.3)	41 (11.7)
Transient corneal haze	3 (0.9)	0 (0.0)	3 (0.9)
Decentered IOL	2 (0.7)	0 (0.0)	2 (0.6)
Dropped IOL	1 (0.3)	0 (0.0)	1 (0.3)
**Total early complications**	**41** (13.3)	**6** (14.3)	**47** (13.4)
No complication	267 (86.7)	36 (85.7)	303 (86.6)
**Late surgical complications**			
Posterior capsular opacity	17 (5.5)	5 (11.9)	22 (6.3)
Chronic uveitis	0 (0.0)	1 (2.4)	1 (0.3)
Strabismus	9 (2.9)	2 (4.8)	11 (3.1)
High IOP	10 (3.2)	0 (0.0)	10 (2.9)
Pupillary membrane	3 (0.9)	1 (2.4)	4 (0.9)
Unexpected high refractive error	4 (1.3)	1 (2.4)	5 (1.4)
**Total late complications**	**43** (13.9)	**10** (23.8)	**53** (15.1)
No complication	265 (86.0)	33 (78.6)	296 (84.6)
**Repeated surgeries**			
IOL repositioning	2 (0.7)	0 (0.0)	2 (0.6)
Removal of IOL from vitreous	1 (0.3)	0 (0.0)	1 (0.3)
Surgical capsulotomy	14 (4.6)	2 (4.8)	16 (4.6)
Membranectomy	2 (0.7)	2 (4.8)	4 (0.9)
IOL exchange	1 (0.3)	1 (2.4)	2 (0.6)
Secondary IOL	7 (2.3)	2 (4.8)	9 (2.6)
Yttrium-Aluminum-Garnet capsulotomy	1 (0.3)	1 (2.4)	2 (0.6)
**Total repeated surgeries**	**28** (9.1)	**8** (19.1)	**36** (10.3)
**Grand total**	**308 (100**)	**42 (100**)	**350 (100**)

IOL, intraocular lens; IOP, intraocular pressure.

### Refractive results

A total of 342/392 (87.2%) eyes that had 1-year follow-up (both bilateral and unilateral) were refracted at 1 month or less postoperatively and 315 (80.4%) eyes were refracted at 1 year postoperatively. A total of 90/342 (26.3%) eyes that were refracted at initial follow-up (1 month or less) had astigmatism and 68/315 (21.6%) eyes that were refracted 1 year after surgery had astigmatism. A total of 32/342 (9.4%) eyes that were refracted at initial follow-up had astigmatism of 1.5 D or more. 34/315 (10.8%) eyes that were refracted 1 year after surgery had astigmatism of magnitude of 1.5 D or more. Against the rule astigmatism of 1.5 D or more rates were 20/90 (22.2%) initially and 16/68 (23.5%) after 1 year. Children with corneal incisions were more likely to have postoperative astigmatism (OR=2.6: p=0.004) than those with scleral at 1 year. Children with refractive error of ≥2.0 D (spherical equivalent) were corrected and any other residual refractive errors were corrected with an appropriate near vision addition. However, children whose parents were unable to pay for spectacles and those under 5 years who had postoperative myopia and were in focus for near were not given spectacles. One hundred and twenty-six out of 196 (64.3%) children with 1-year follow-up were given glasses. One hundred and nine out of 125 (87.2%) children given glasses were wearing them at follow-up. Refractive outcomes (spherical equivalent) are shown in table 3.

**Table 3:** Frequency of postoperative refractive error (spherical equivalent) (n=405)

Postoperative visit	Refractive error (D), frequency (%)						
	−0.50 to +1.00 D (emmetropia)	>+1.00 to +4.00 D (low to moderate hypermetropia)	>+4.00 D (high hypermetropia)	−4.00 to −0.75 D (low to moderate myopia)	>−4.00 D (high myopia)	Aphakia	Missing
First	83 (24.3)	75 (21.9)	41 (11.9)	70 (20.5)	15 (4.4)	59 (17.3)	63
At 1 year	74 (23.5)	80 (25.4)	24 (7.6)	82 (26.0)	22 (6.9)	33 (10.5)	90

D, diopters.

**Figure 1:**
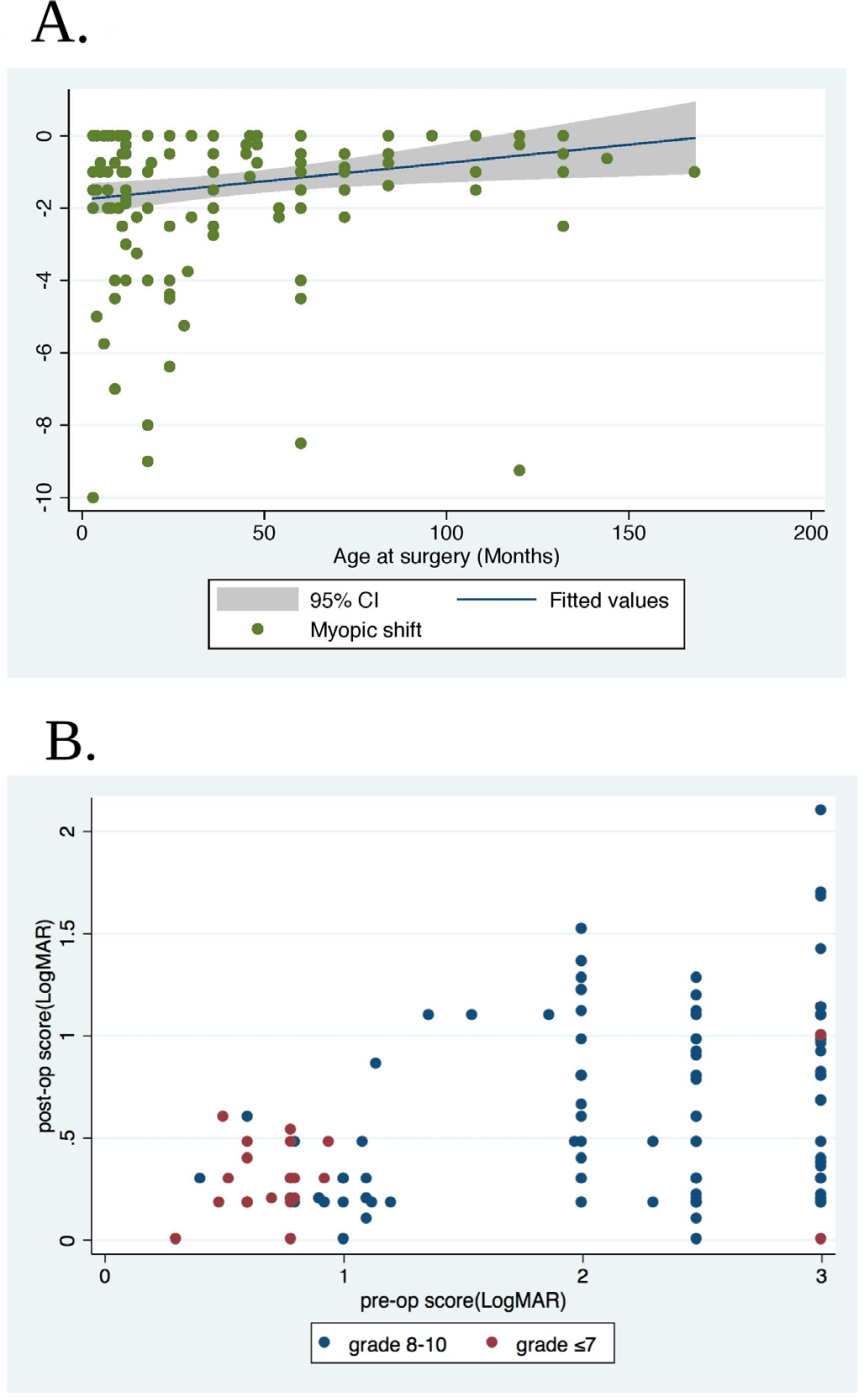
(A) Myopic shift between early postoperative and 1-year refraction (dioptres) versus age at surgery (months). (B) Preoperative versus postoperative Visual Acuity Score.

Children with postoperative hypermetropia ≥3 DS were younger (p=<0.001) and had shorter eyes (p<0.001) than those without. During the first postoperative year, there was a mean myopic shift magnitude of 1.34 D (SD 1.9; range 0.00–10.00D; n=185). For every unit (month) decrease in age at surgery there was increase in mean myopic shift by 0.018 D; 95% CI 0.013 to 0.021; (p<0.001). Myopic shift vs age at surgery is shown in figure 1A. Mean final refractive error (spherical equivalent) postoperative was smaller (p<0.001) in patients who had keratometry readings compared with those who did not.

### Visual results

#### Bilateral cataract

A total of 154 of 177 (87%) bilateral children had a quantitative postoperative visual acuity assessment after 1-year follow-up. Eighty-three (53.9%) of these achieved visual acuity of 0.48 logMAR or better in their better eye; 39 (25.3%) had best-corrected vision between 0.50–1.00 logMAR; 24 (15.6%) had best-correct vision 1.00–1.3 logMAR and 8 (5.2%) were worse than logMAR 1.3, 1 year postoperatively. The proportion of better eyes with 1-year best-corrected visual acuity worse than 1.00 logMAR was lower (p<0.001) for less dense (grade <5) cataracts (3.12%) compared with denser (grade 8–9) cataracts (84.38%). 65/103 (63.1%) who had Teller acuity test at follow-up achieved corrected visual acuity of 0.48 logMAR or better in their better eye and 29 out of 51 (39.2%) children who had recognition acuities achieved best-corrected visual acuity of 0.48 logMAR or better in their better eye. Preoperative and postoperative visual acuity results for children with bilateral cataracts are shown in figure 1B.

Of 68, 22 (32.3%) children operated below 2 years achieved a corrected visual acuity of 0.48 logMAR or better in the better eye, compared with 61 out of 86 (70.9%) children operated at the age of 2 or older (p<0.001).

Of 177, 169 (95.5%) had follow-up at 3 months. Of 177, 37 (20.9%) children achieved visual acuity of 0.48 logMAR or better in the better eye at 3 months follow-up which significantly increased to 83 out of 154 (53.9%) children at 1-year follow-up (p<0.001). Independent predictors of poor visual outcome (worse than 0.48 logMAR in the better eye) are shown in table 4.

**Table 4:** Factors associated with poor visual outcome among children with bilateral cataract (defined as best corrected visual acuity of less than 0.48 in the better eye at 1 year) (n=154)

Variables	Total (n=154)	Poor outcome (n=72)	Unadjusted analysis			Adjusted analysis		
			OR	(95% CI)	P value	OR	(95% CI)	P value
Age at surgery		n (%)						
≤24 months	78	52 (66.7)	1.00	–	–	1.00		
>24 months	76	20 (26.3)	0.18	0.08 to 0.38	**<0.001**	0.39	0.14 to 1.10	0.076
Delayed presentation/lag time								
≤3 months	28	15 (53.6)	1.00	–	–			
>3 to ≤12 months	57	31 (54.4)	1.03	0.41 to 2.57	0.945			
>12 months	65	23 (35.4)	0.47	0.29 to 3.32	0.104			
Missing	4	3 (75.0)						
Preoperative blindness								
No	38	4 (10.5)	1.00	–	–	1.00		
Yes	116	68 (58.6)	12.04	3.58 to 40.51	**<0.001**	14.65	2.21 to 97.20	**0.005**
Preoperative nystagmus/strabismus								
No	50	7 (14.0)	1.00	–	–	1.00		
Yes	105	65 (61.9)	10.24	3.75 to 27.97	**<0.001**	9.22	2.66 to 31.97	**<0.001**
Microphthalmos								
No	119	48 (40.3)	1.00	–	–	1.00		
Yes	28	20 (71.4)	3.69	1.46 to 9.37	**0.003**	1.12	0.29 to 4.29	0.872
Missing	7	4 (57.1)						
Aphakia								
No	128	50 (39.1)	1.00	–	–	1.00		
Yes	26	22 (84.6)	8.58	2.59 to 28.46	**<0.001**	5.32	1.05 to 26.97	**0.043**
Astigmatism								
No	109	58 (45.9)	1.00	–	–	1.00		
Yes	33	11 (33.3)	0.44	0.19 to 1.01	**0.046**	0.91	0.29 to 2.79	0.865
Missing	12	3 (25.0)						
Cataract grade								
Grade ≤7	22	3 (13.6)	1.00			1.00		
Grade 8–10	132	69 (52.3)	6.90	1.86 to 25.92	**<0.001**	0.45	0.03 to 6.07	0.548
Refractive error								
Myopia (≥−1.5 D)	38	14 (36.8)	1.00					
Hypermetropia (>1.0 D)	43	21 (48.8)	1.64	0.66 to 4.03	0.280			
Missing/emmetropia	73	37 (50.7)						
High myopia (>−4.0 D)								
No	108	43 (39.9)	1.00					
Yes	10	4 (40.0)	1.01	0.27 to 3.80	0.991			
Missing	36	25 (69.4)						
High hypermetropia (>4.0 D)								
No	103	38 (39.9)	1.00					
Yes	15	9 (60.0)	2.57	0.83 to 7.91	0.089			
Missing	36	25 (69.4)						
Early postoperative complications								
No	125	54 (43.2)	1.00					
Yes	29	18 (62.1)	2.15	0.93 to 4.99	0.07			
Late postoperative complications								
No	132	63 (47.7)	1.00					
Yes	22	9 (40.9)	0.76	0.30 to 1.90	0.55			
Keratometry performed								
No	50	23 (46.0)	1.00					
Yes	104	49 (47.1)	1.05	0.53 to 2.06	0.89			
Systemic problem								
No	97	43 (44.3)	1.00					
Yes	57	29 (50.9)	1.30	0.67 to 2.51	0.43			
Anaemia								
No	76	30 (39.5)	1.00			1.00		
Yes	74	41 (55.4)	1.91	0.98 to 3.69	**0.05**	0.86	0.32 to 2.31	0.765
Missing	4	1 (25.0)						

Bold type denotes statisticaly significant values.

#### Unilateral cataract

Forty-two of 51 (82.4%) had quantitative acuity at 1-year follow-up. Fourteen of42 (33.3%) achieved visual acuity of 0.48 logMAR or better; 10 (23.8%) had vision between <0.48 and 1.00 logMAR; 11 (26.2%) had vision <1.00–1.3 logMAR; and 7 (16.7%) were blind, 1 year postoperatively. Forty-six 0 f 51 (90.2%) were blind preoperatively. Only surgery at <24 months of age predicted poor visual outcome in a univariate analysis (OR=0.09: p=0.025).

There were no significant differences between children who attended for follow-up at 1 year and non-attenders, in terms of the factors, which predicted visual outcome for bilateral and unilateral cases.

## Discussion

This is the first prospective study reporting outcomes of childhood cataract surgery from East Africa. The majority of children with bilateral cataracts (and 1-year follow-up) achieved better eye vision of at least 0.48 logMAR. This proportion was better than some retrospective reports from SSA^6 16 17^ possibly because of longer follow-up in this study, as visual outcomes after cataract surgery in children tend to improve with time. On the other hand, it was a slightly smaller proportion than reported in Tanzania^5^ where 62% of children operated achieved vision equal or better than 0.48 logMAR in the better eye. However, the proportion of children with follow-up in that study was much poorer which would have likely biased the results. In our study, over 50% of the bilateral children had ‘normal’ vision after surgery, by WHO classification despite 75% blindness before surgery (only 5.2% postoperatively).

Half the children had a lag time of 1 year or more. This is slightly less than that reported in 2005^9^ where the 69% of children had such delay, possibly due to introduction of child eye-health awareness programmes aimed to address the barriers to child eye health services in the East African region through ‘Seeing is Believing’.^18^

In our study, nystagmus was present in nearly half, higher than that previous reports from the region^6^ indicating that a high proportion of our sample had congenital cataracts.

The proportion of children with congenital and developmental cataract who have low body weights and low haemoglobin levels during surgery were estimated to be 68.4% and 48.4%, respectively, more common in bilateral cases. Low haemoglobin levels in congenital and developmental cataracts have previously been reported^19^ but information on low body weight in children operated for congenital and developmental cataracts has not been shown before and warrants further investigation on aetiology of developmental cataract in children and its effects on general health.

Delayed presentation did not predict poor visual outcome. This is surprising because in children with cataract who are visually deprived for longer period of time are thought to be more amblyopic. However, our data showed that high-grade cataracts are more likely to present much earlier than low-grade cataracts, perhaps negating the effect of lag time on vision. On multivariate analysis, preoperative blindness was found to be significant predictor of poor outcome (p=0.004) similar to previous reports from Tanzania and Kenya, suggesting that cataract density could be a more important determinant of deprivation amblyopia than delay in presentation over a certain period of time. Surgery at younger age predicted poor visual outcome. This has also been reported in France^20^ where the best visual outcomes were obtained in late surgery (older children≥7 months) compared with early surgery (younger children <7 months). This can be explained by the fact that younger children are more likely to have congenital cataract with worse amblyopia and nystagmus.

In this study, aphakia also predicted poor visual outcome. This was also seen in France^20^ probably due to the fact that eyes with other ocular abnormalities (eg, microphthalmos) hence poor visual prognosis were more likely to be left aphakic. In contrast, large multicentre studies in the UK and USA where contact lenses are readily available, showed no visual benefit from pseudophakia over aphakia.^21 22^

There was a change of surgical technique during the study, from the standard 3 mm superior scleral tunnel incision to 3 mm (usually sutureless) superior corneal incision. The aim was to reduce the rate of second general anaesthetic (GA) procedures (since nylon sutures need to be removed) and rate of astigmatism. Authors found that a well-constructed 3 mm superior corneal incision that barely nicks the limbal blood vessels and injecting intracameral air at the end of surgery with additional hydro inflation of the paracenteses in children above 1 year was sufficient; there were no wound leaks, endophthalmitis or shallow anterior chambers and repeat GA procedures were reduced, 10% vs 28% in a previous study with sutured paracenteses.^9^

There was a low rate of initial astigmatism of 1.5 D or more at 2 weeks follow-up and after 1 year of follow-up. However, the majority was against the rule and an absorbable suture for the corneal wound might reduce this tendency.

Forty-three out of 71 (60.6%) eyes of children ≤1 year had hyperopia of ≥2.0 D compared with 25 out of 110 (22.7%) eyes of children greater than 1 year (p<0.001). This is probably because of deliberate under correction of younger children. Three-piece lenses were preferred in most difficult surgeries, younger children, microphthalmic eyes and sulcus fixation. These factors could therefore explain the link between three-piece lenses with more uveitis.

## Conclusion

Good visual results are achievable in this setting despite long lag times. Preoperative blindness and preoperative nystagmus/strabismus were significant predictors of poor visual outcome suggesting that cataract density determines density of amblyopia. Anaemia and low body weight seem to coexist in large proportion of children with non-traumatic cataracts in Tanzania, warranting further investigation. Sutureless surgery seems to be safe in children over 1 year old.
