# Supplementary material for: Presentation, surgery and 1-year outcomes of childhood cataract surgery in Tanzania
Source: Br J Ophthalmol. 2020 Jun 10;105(3):334–40. doi: 10.1136/bjophthalmol-2020-316042 (PMC7907562; doi:10.1136/bjophthalmol-2020-316042)
Supplement: Supplementary data [file bjophthalmol-2020-316042supp001.pdf]

Supplementary table 1: Associated motility disturbances by laterality (N=228)

Associated motility disturbances	Total n (%)	Laterality		P-value
		Bilateral n (%)	Unilateral n (%)	
Nystagmus without strabismus	72 (31.6)	68 (38.4)	4 (7.8)	**<0.001**
Strabismus without nystagmus	41(17.9)	19 (10.7)	22 (43.1)	**<0.001**
Nystagmus and strabismus	40 (17.5)	34 (19.2)	6 (11.8)	0.109
Neither nystagmus nor strabismus	75(32.9)	56(31.6)	19(37.3)	0.226
**Total**	**228 (100)**	**177 (100)**	**51(100)**	

Supplementary table 2: Distribution of lag time according to laterality and gender (n=228)

	Laterality								
	Bilateral				Unilateral				
	Gender				Gender				
Lag time (months)	Male n (%)	Female n (%)	**Total**	Cumm Freq.	Male n (%)	Female n (%)	**Total**	Cumm Freq.	**Grand Total**
≤3	23(20.7)	12(18.2)	**35(19.8)**	**19.8**	10(33.3)	7(33.3)	**17(33.3)**	**33.3**	**52(22.8)**
4-6	10(9.0)	5(7.6)	**15(8.5)**	**28.3**	3(10.0)	1(4.8)	**4(7.8)**	**41.1**	**19(8.3)**
7-12	29(26.1)	21(31.8)	**50(28.2)**	**56.5**	3(10.0)	4(19.0)	**7(13.7)**	**54.8**	**57(25.0)**
13-18	4(3.6)	2(3.0)	**6(3.4)**	**59.9**	3(10.0)	2(9.5)	**5(9.8)**	**64.6**	**11(4.8)**
19-24	13(11.7)	11(16.7)	**24(13.6)**	**73.5**	3(10.0)	3(14.3)	**6(11.8)**	**76.4**	**30(13.2)**
25 and above	28(25.2)	12(18.2)	**40(22.6)**	**96.1**	6(20.0)	4(19.0)	**10(19.6)**	**96.0**	**50(21.90**
Missing	4(3.6)	3(4.5)	**7(3.9)**	**100.0**	2(6.7)	0(0.0)	**2(3.9)**	**100.0**	**9(3.9)**
**Total**	**111(100)**	**66(100)**	**177(100)**		**30(100)**	**21(100)**	**51(100)**		**228(100)**
